# Intrahepatic Cholangiocarcinoma Associated with High Procalcitonin, Hypercalcemia, Polycythemia and Leukocytosis

**DOI:** 10.7759/cureus.6587

**Published:** 2020-01-07

**Authors:** Sreenath Meegada, Richard Eisen, Gregory Coons, Rajanshu Verma

**Affiliations:** 1 Internal Medicine, The University of Texas Health Science Center/Christus Good Shepherd Medical Center, Longview, USA; 2 Pathology, Banner Thunderbird Medical Center, Glendale, USA; 3 Gastroenterology, University of Tennessee Health Science Center, Memphis, USA

**Keywords:** cholangiocarcinoma, procalcitonin, hypercalcemia, polycythemia, leukocytosis

## Abstract

Intrahepatic cholangiocarcinomas or bile duct cancers comprise approximately 10-20% of all cholangiocarcinomas and may present with right upper quadrant pain, weight loss, liver enzyme abnormalities or they may be completely asymptomatic and be picked incidentally on routine abdominal imaging. Typically, hepatocellular carcinomas have been associated with various paraneoplastic syndromes such as hypercalcemia, erythrocytosis, hypoglycemia, diarrhea and skin changes though paraneoplastic syndromes in the setting of cholangiocarcinoma do occur as well. Cholangiocarcinomas are usually associated with dermal paraneoplastic syndromes (Sweet syndrome, porphyria cutanea tarda, acanthosis nigricans, necrotic migratory erythema, erythema multiforme, bullous pemphigoid), hypercalcemia, leukocytosis and limbic encephalitis. We present a case of an 80-year-old man with intrahepatic cholangiocarcinoma associated with high procalcitonin levels in the absence of infection, paraneoplastic syndromes of hypercalcemia, polycythemia and leukocytosis in the same individual. This constellation of symptoms, to the best of our knowledge, has not been previously reported in the scientific literature.

## Introduction

Intrahepatic cholangiocarcinoma (ICC) is a very rare gastrointestinal malignancy that accounts for 3% of entire gastrointestinal malignancies [1]. ICC has distinct epidemiology, and clinical course compared to hilar and extrahepatic ICCs and comprises less than 10-20% of all cholangiocarcinomas [2]. Cholangiocarcinomas have been anatomically classified into three types based on the site of origin: 1. intra-hepatic (within liver up to common hepatic duct), 2. perihilar (origin of common hepatic duct to joining of cystic duct with common bile duct), 3. distal extrahepatic (origin of common bile duct to the ampulla of Vater) [2]. Complex interaction between cancer and immune system is seen in paraneoplastic syndromes [3]. Our aim is to present this patient with ICC, characterized by rare paraneoplastic syndromes.

## Case presentation

An 80-year-old Caucasian man presented to the emergency department (ED) with one-week history of ankle swelling and abdominal pain. Per family members, the patient was confused, having memory loss, increased urinary frequency, cough, mild dyspnea and generalized weakness. He had a prior history of hypertension, dyslipidemia, chronic kidney disease stage III, benign prostatic hyperplasia, transitional cell carcinoma of the urinary bladder for which he underwent transurethral resection of bladder tumor. His prescription medications included tamsulosin, benazepril, simvastatin and a multivitamin. He denied any intake of calcium supplements, antacids or vitamin D. He was a former alcoholic (quit 10 years ago) and an ex-smoker (quit 15 years ago) and denied any illicit drug use. He was recently discharged from the hospital a week ago when he was admitted for acute kidney injury, secondary to dehydration.

All his initial vital signs in the emergency department were within normal limits and physical examination was unremarkable with the exception of 2+ pitting pedal edema bilaterally up to mid-shins. Serum chemistries showed a white blood cell count (WBC) 15,600/mm^3^ (normal: 4,000-11,000), neutrophil count 12,900/mm^3^ (normal: 1500-8,000), monocytes 1300/mm^3^ (normal: 200-1000), hemoglobin 17.4 g/dl (normal: 13.5-17.0), red blood cell 5.78 million/mm^3^ (normal: 4.3-6.0), platelets 145,000/mm^3^ (normal: 150,000-450,000), blood urea nitrogen 31 mg/dl (normal: 7-20), creatinine 1.76 mg/dl (normal: 0.6-1.2), calcium 12.2 mg/dl (normal: 8.5-10.2), albumin 3.1 g/dl (normal: 3.5-5.5) [corrected calcium 12.9 mg/dl], total bilirubin 1.5 mg/dl (normal: 0.1-1.2), aspartate aminotransferase (AST) 73 IU/L (normal: <40), alanine aminotransferase (ALT) 33 IU/L (normal: <56), alkaline phosphatase (ALP) 218 IU/L (normal: 44-147), NT-proBNP 638 pg/ml (normal: <450) and serum procalcitonin 35.6 ng/ml (normal: <0.5).

Chest radiograph done in emergency department showed bilateral infrahilar and basilar subsegmental atelectasis though early infiltrate versus aspiration could not be ruled out. Ventilation-perfusion nuclear medicine scan showed low probability of pulmonary embolism. The patient was empirically started on piperacillin-tazobactam by ED physician for possible healthcare-associated pneumonia and admitted to the hospital for further care.

Suspecting the possibility of congestive heart failure, the admitting physician started patient on intravenous furosemide, ordered an echocardiogram and added intravenous vancomycin for empiric coverage as well. Echocardiogram showed a normal ejection fraction of 60% with no valvular abnormalities. Nephrology service was consulted by the admitting physician for management of hypercalcemia. Nephrologist ordered studies to evaluate the cause of hypercalcemia, discontinued furosemide and gave calcitonin to treat hypercalcemia. Investigative workup for hypercalcemia revealed parathyroid hormone level < 6 pg/ml (normal: 15-65), 25-hydroxy vitamin D 45 ng/ml (normal: 30-100), 1, 25-dihydroxy vitamin D 17 pg/ml (normal: 18-72), angiotensin converting enzyme level 11 U/L (normal: 9-67), 24-hour urinary calcium excretion 153 mg/dl (normal: 50-300), normal IgA, IgG, IgM levels, serum protein electrophoresis showed hypoalbuminemia without any evidence of monoclonal protein, serum and urine immunofixation was negative. Ordered serum parathyroid hormone-related peptide (PTHrP) revealed elevated levels of 62 pg/ml (normal: 14-27 pg/ml). As hypercalcemia did not respond to calcitonin, the patient was given intravenous pamidronate (twice). As his leukocytosis continued to worsen (19,100/mm^3^) along with procalcitonin (38.15 ng/ml) despite being on broad-spectrum antibiotics for one-week, infectious diseases service was consulted and leukemia/lymphoma flow cytometry panel, peripheral blood smear was ordered to evaluate for hematologic malignancy. Infectious disease physician discontinued vancomycin and ordered Indium-111 labeled whole body WBC scan which showed no abnormal areas of increased tracer uptake to suggest a focus of infection. Leukemia/lymphoma flow cytometry was negative for any specific phenotypic abnormality. Peripheral blood smear showed presence of neutrophilia without any evidence of malignant leukocytes.

**Figure 1:**
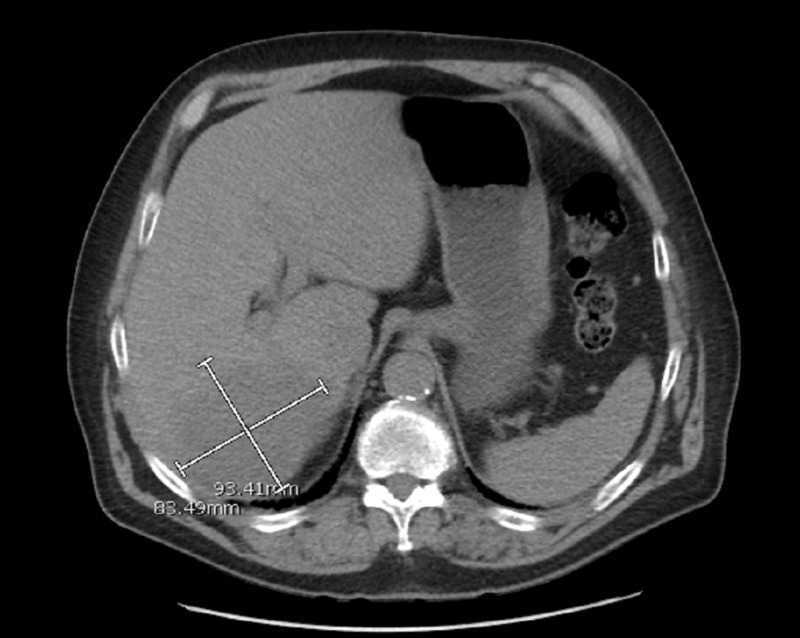
CT abdomen/pelvis (axial view) showing a hypodense mass in the right posterior lobe measuring 9.3 x 8.3 cm in size (see marked scale)

Meanwhile, the patient went in to acute hypoxic respiratory distress with wheezing and so pulmonary service was consulted which transferred the patient to ICU, started him on intravenous methylprednisolone and initiated non-invasive positive pressure ventilation (bi-level positive airway pressure - BiPAP). As PTHrP had come back elevated, the patient underwent a CT scan of chest, abdomen and pelvis, which showed a hypodense mass in the right posterior lobe of liver measuring 9.3 cm x 8.3 cm in size with presence of moderate abdominal and severe pelvic ascites (Figure 1). The patient underwent paracentesis with removal of 2.6 L of straw-colored fluid. Ascitic fluid did not show any malignant cells or evidence of spontaneous bacterial peritonitis. Ultrasound of the liver showed a 10.9-cm mass in the right lobe of liver along with portal vein occlusion. Hematology and oncology service was then consulted. Oncologist did not recommend anticoagulation for portal vein occlusion and ordered tumor markers evaluation which showed alpha-fetoprotein 14.7 ng/ml (normal: <8.7), CA 19-9 2907 U/ml (normal: < 35), carcinoembryonic antigen (CEA) 5.2 ng/ml (normal: <3.4). A possibility of hepatocellular carcinoma was discussed with the patient and he was scheduled to undergo CT-guided liver biopsy. Broad spectrum antibiotics were discontinued after 10 days of administration as they neither lowered procalcitonin nor WBC count. Blood, urine and other body fluid cultures remained negative during the entire hospital stay. Either partly because of being on intravenous steroids and/or due to underlying disease process, patient’s WBC count peaked at 40,100/mm^3^ during this hospital stay. Though he presented with polycythemia, his peak hemoglobin and hematocrit during admission was 19.7 g/dl and 57%. Serum erythropoietin level was found to be elevated 21.4 mIU/ml (normal: 2.6-18.5).

**Figure 2:**
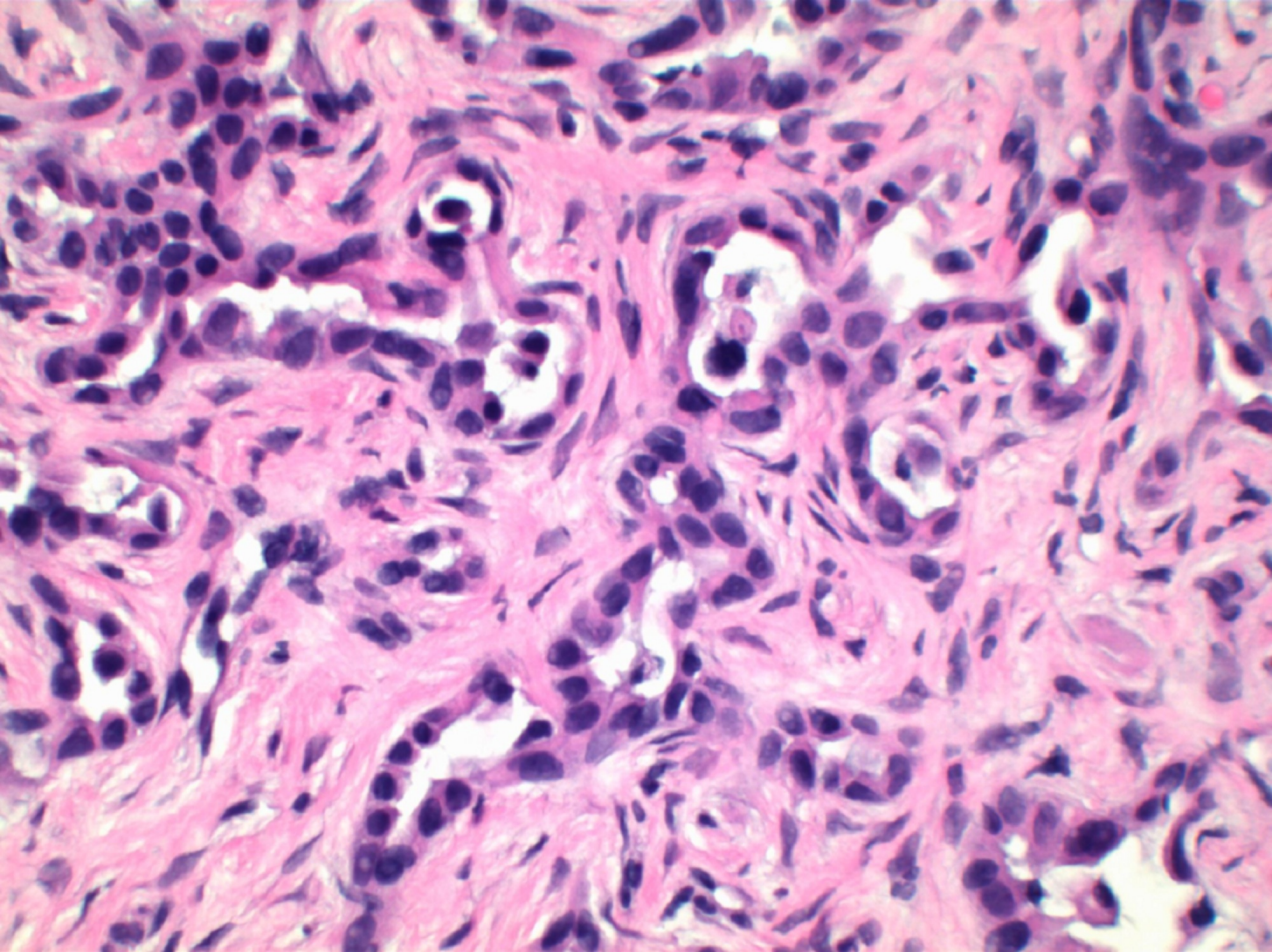
H&E stain (100x magnification) showing moderately differentiated adenocarcinoma reported as primary intrahepatic cholangiocarcinoma based on immunohistochemical stains

**Figure 3:**
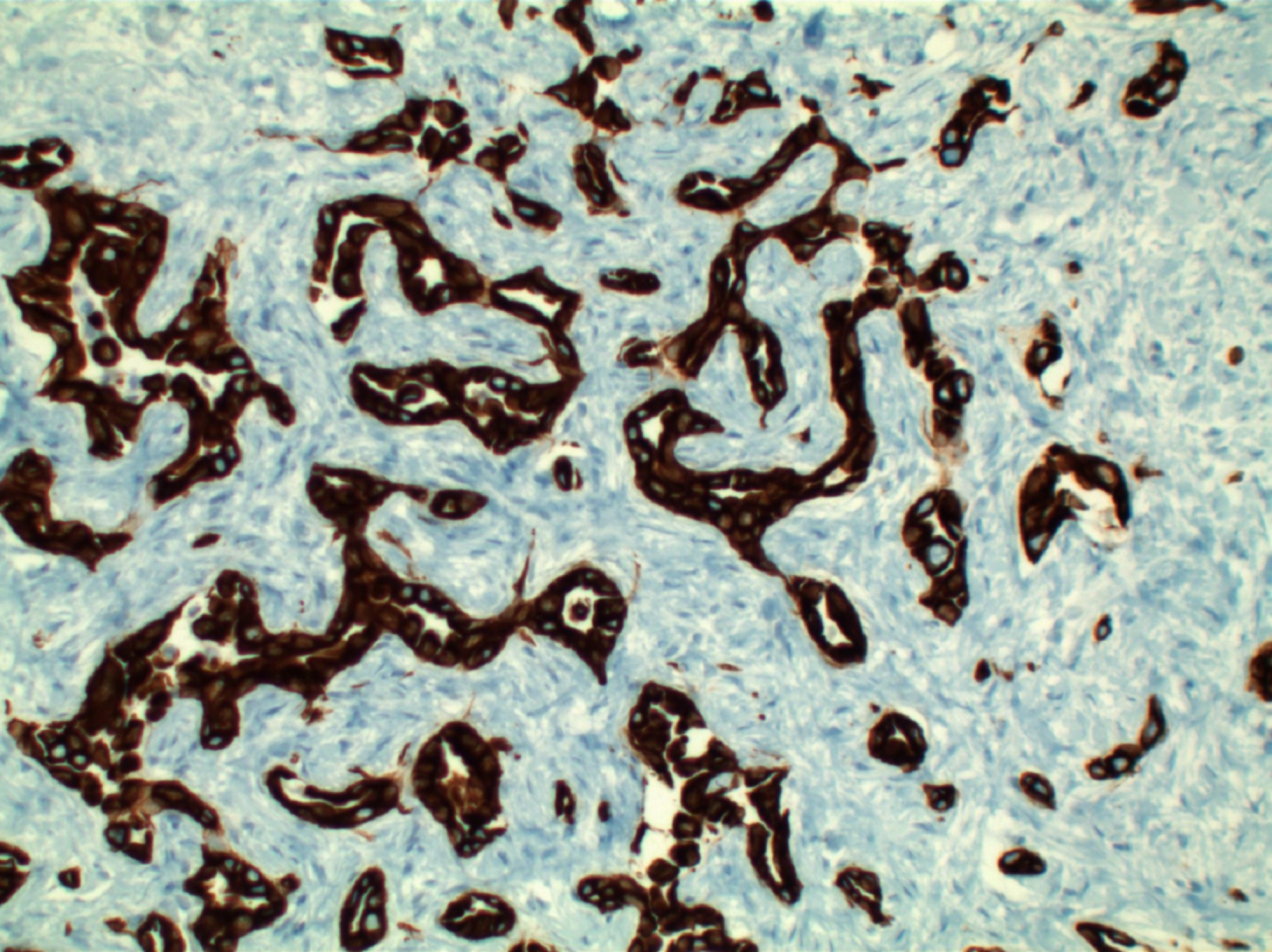
CK7 staining of bile ducts with sparing of hepatocytes in this intra-hepatic cholangiocarcinoma

**Figure 4:**
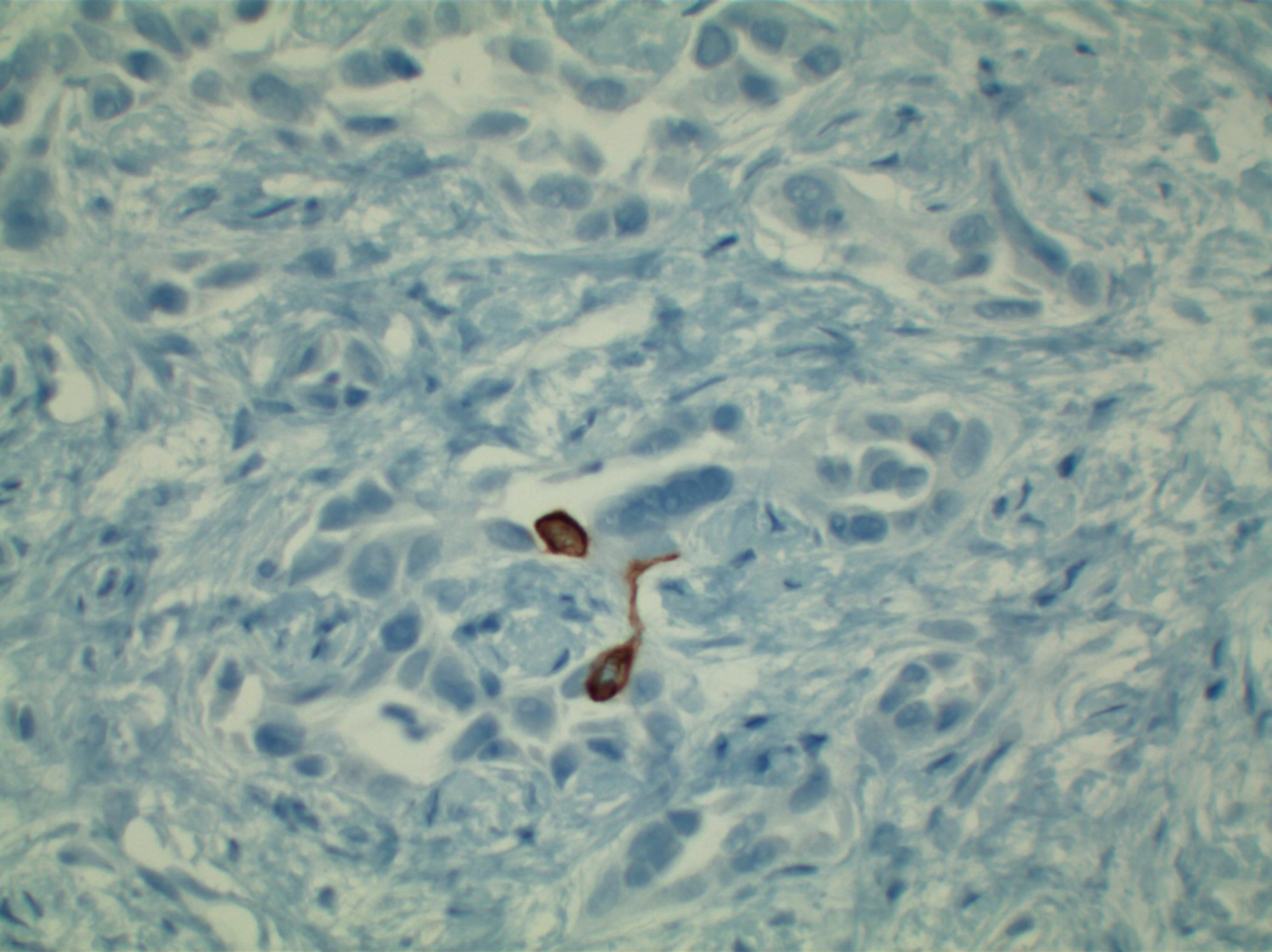
Predominantly negative CK20 staining helping rule out metastatic colon adenocarcinoma in this specimen

**Figure 5:**
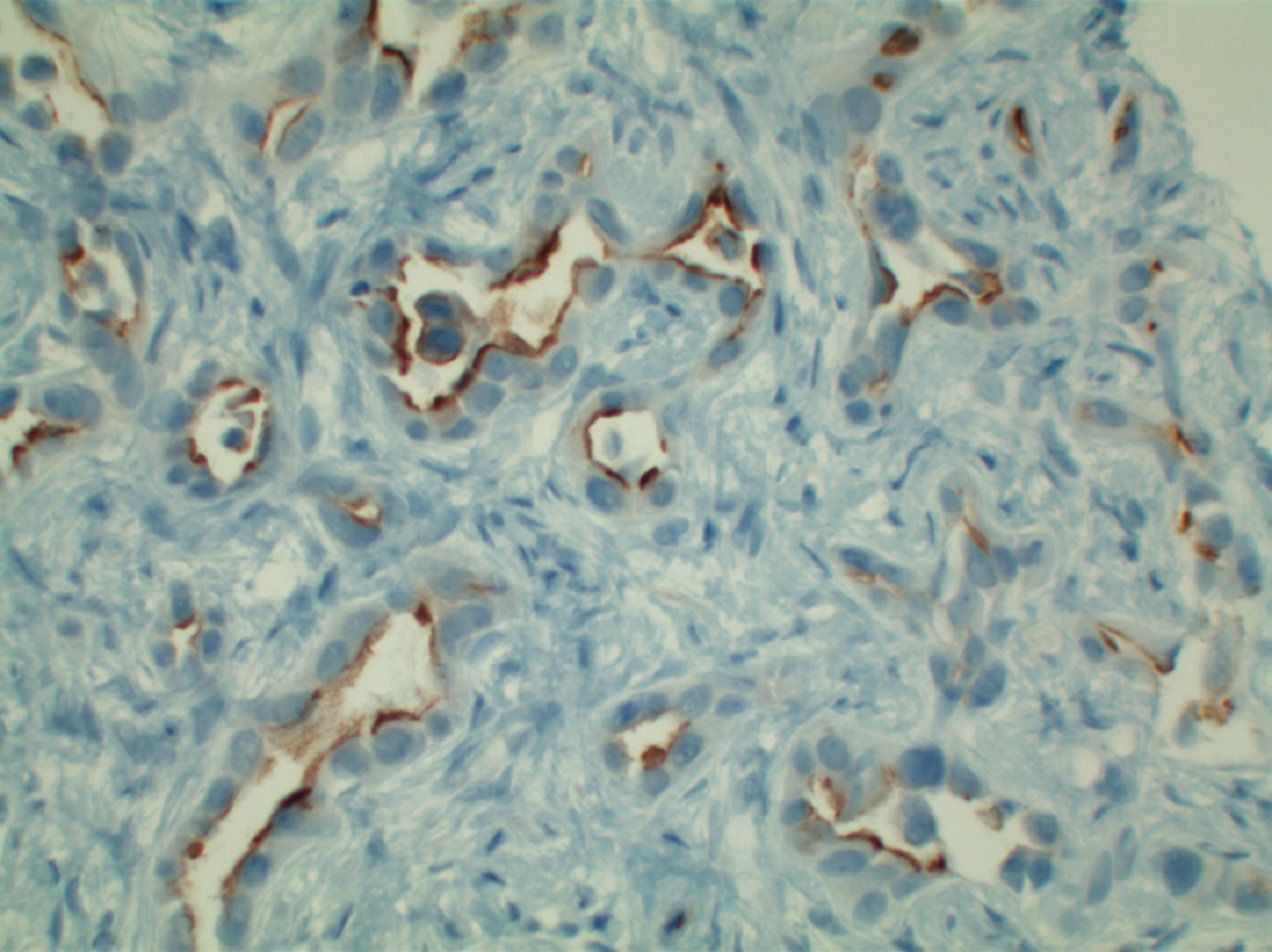
Positive villin stain showing hepatic bile ducts in this intra-hepatic cholangiocarcinoma specimen

**Figure 6:**
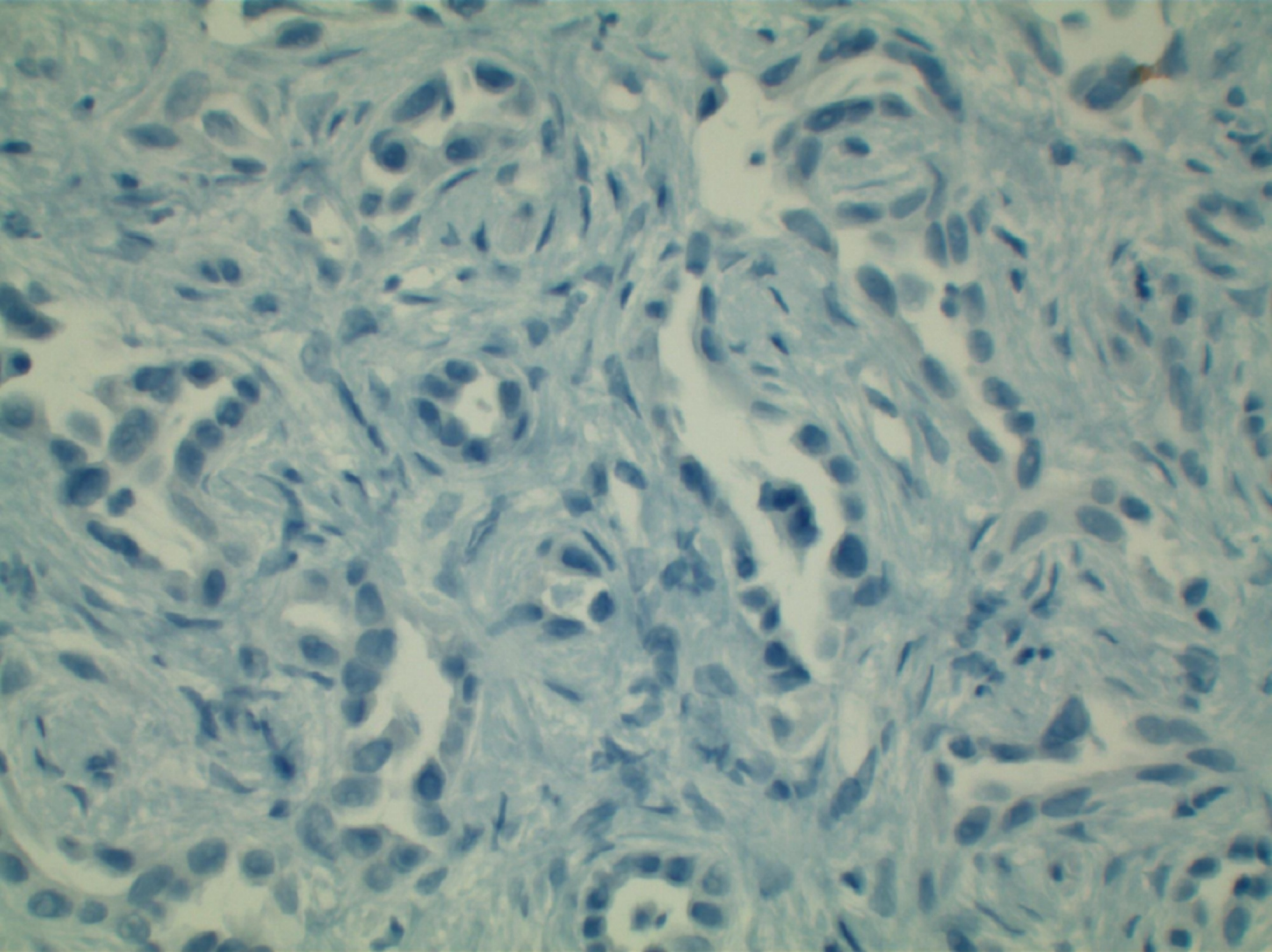
Negative CDX-2 stain distinguishing this specimen from metastatic adenocarcinoma of intestinal origin

Patient’s liver profile worsened during his ICU stay with serum bilirubin escalating up to 12 mg/dl, ALP 470 IU/L, AST 429 IU/L, ALT 362 IU/L, international normalized ratio (INR) 2.8, albumin 1.8. MRI liver/magnetic resonance cholangiopancreatography (MRCP) abdomen was ordered which did not show any evidence of biliary obstruction. Viral hepatitis panel came back negative as well. Liver biopsy revealed moderately differentiated ductal adenocarcinoma of hepatobiliary origin reported as primary intrahepatic cholangiocarcinoma under current clinical settings (Figure 2). Immunohistochemical stains revealed CK Oscar(+), CK7(+) (Figure 3), CK20(-) (Figure 4), Villin(+) (Figure 5), CDX2(-) (Figure 6), TTF-1(-), Napsin A(-), PSA(-) and NKX3.1(-).

Oncology service recommended no chemotherapy at the moment as the patient was thought to be too frail. Three weeks after his admission to the hospital, his blood pressure dropped in ICU requiring initiation of norepinephrine infusion. A temporary hemodialysis catheter was inserted and sustained-low efficiency dialysis (SLED) was initiated as well. Given his dismal prognosis in the setting of malignancy and multi-organ failure, a detailed discussion was held with the patient and his family members when it was decided to proceed with comfort care options and hospice. The patient was discharged to hospice the following day.

## Discussion

Paraneoplastic syndromes are rarely seen in ICC, compared to hepatocellular carcinomas. Thus, our present case is one of such a few notable conditions. Contrary, cholangiocarcinomas are usually associated with cutaneous paraneoplastic syndromes like sweet syndrome, porphyria cutanea tarda, acanthosis nigricans, erythema multiforme, subacute cutaneous lupus erythematosus [3].

Hypercalcemia in malignancy may arise from three different mechanisms: Humoral hypercalcemia from secretion of parathyroid hormone-related peptide (PTHrP), osteolysis from osteoclast activation and tumor production of calcitriol. Humoral hypercalcemia is the most common cause and is seen in up to 80% of cancer patients [4]. Osteolytic metastases are the next common cause of hypercalcemia and may be present in up to 20% of patients [3]. Hypercalcemia is a rare paraneoplastic manifestation of cholangiocarcinoma [5]. Humoral hypercalcemia in cholangiocarcinoma is a predictor of disease burden and poor prognosis [6].

As observed from our patient's findings, elevated procalcitonin levels have never been previously reported in patients with cholangiocarcinoma in absence of concurrent infection. Procalcitonin is a serum biomarker that helps to distinguish bacterial infections from other infectious causes or inflammation. Procalcitonin is synthesized within the thyroid neuroendocrine cells and is then released in its final form, which is calcitonin [7]. Systemic inflammation in body sets in procalcitonin synthesis in all tissues by endotoxins, cytokines, and interleukins released during bacterial infection [8]. Serum procalcitonin levels are rarely elevated in cancers, except medullary thyroid cancers, neuroendocrine tumors, and in lung cancers with neuroendocrine components or multiple metastases [9-11].

Leukocytosis with neutrophil predominance is an extremely rare paraneoplastic syndrome associated with patients with cholangiocarcinoma. Granulocyte colony-stimulating factor (G-CSF) stimulates the proliferation of hematopoietic progenitor cells of neutrophil granulocytes. It is mainly produced by monocytes, macrophages, and lymphocytes when activated by tumor necrosis factor-alpha, lipopolysaccharide, and interferon-gamma [12]. G-CSF can also be produced by cancer cells, which upregulates the progression of the tumor [13]. Rarely, as presented by this patient cholangiocarcinoma can present with paraneoplastic leukemoid reaction (PLR) [14].

Polycythemia is more common in hepatocellular carcinoma because of erythropoietin secretion from cancer cells [15]. It is rarely reported in patients with cholangiocarcinoma. Polycythemia is also seen in other tumors like renal cell carcinoma, hemangioblastoma, uterine leiomyomas, and pheochromocytoma [16-20].

## Conclusions

Our patient was diagnosed with ICC on biopsy, along with very rare combination of paraneoplastic syndromes which include hypercalcemia, leukocytosis, polycythemia/erythrocytosis, and elevated serum procalcitonin. ICC with hypercalcemia and leukocytosis is rare, and ICC with elevated serum procalcitonin and polycythemia has never been reported to the best of our knowledge. This case report helps spread awareness among readers that intrahepatic cholangiocarcinoma may be associated with various types of paraneoplastic syndromes and physicians taking care of such patients should be cognizant of such rare presentation of this cancer.
